# Trends in national and subnational wealth related inequalities in use of maternal health care services in Nepal: an analysis using demographic and health surveys (2001–2016)

**DOI:** 10.1186/s12889-020-10066-z

**Published:** 2021-01-04

**Authors:** Vishnu Prasad Sapkota, Umesh Prasad Bhusal, Kiran Acharya

**Affiliations:** 1 Central Department of Public Health (CDPH), Institute of Medicine (IOM), Tribhuvan University, Kathmandu, Nepal; 2 Melbourne School of Population and Global Health, The University of Melbourne, Melbourne, Victoria Australia; 3 New ERA, Rudramati Marga, Kalopul, Kathmandu, 44621 Nepal

**Keywords:** Maternal health, Inequality, Subnational, Nepal, DHS

## Abstract

**Background:**

Maternal health affects the lives of many women and children globally every year and it is one of the high priority programs of the Government of Nepal (GoN). Different evidence articulate that the equity gap in accessing and using maternal health services at national level is decreasing over 2001–2016. This study aimed to assess whether the equity gap in using maternal health services is also decreasing at subnational level over this period given the geography of Nepal has already been identified as one of the predictors of accessibility and utilization of maternal health services.

**Methods:**

The study used wealth index scores for each household and calculated the concentration curves and indexes in their relative formulation, with no corrections. Concentration curve was used to identify whether socioeconomic inequality in maternity services exists and whether it was more pronounced at one point in time than another or in one province than another. The changes between 2001 and 2016 were also disaggregated across the provinces. Test of significance of changes in Concentration Index was performed by calculating pooled standard errors. We used R software for statistical analysis.

**Results:**

The study observed a progressive and statistically significant decrease in concentration index for at least four antenatal care (ANC) visit and institutional delivery at national level over 2001–2016. The changes were not statistically significant for Cesarean Section delivery. Regarding inequality in four-ANC all provinces except Karnali showed significant decreases at least between 2011 and 2016. Similarly, all provinces, except Karnali, showed a statistically significant decrease in concentration index for institutional delivery between 2011 and 2016.

**Conclusion:**

Despite appreciable progress at national level, the study found that the progress in reducing equity gap in use of maternal health services is not uniform across seven provinces. Tailored investment to address barriers in utilization of maternal health services across provinces is urgent to make further progress in achieving equitable distribution in use of maternal health services. There is an opportunity now that the country is federalized, and provincial governments can make a need-based improvement by addressing specific barriers.

## Background

Maternal health affects the lives of many women and children globally every year. Global maternal mortality remains unacceptably high as a consequence of pregnancy and childbirth-related problems [1]. Two-thirds of the countries had a maternal mortality ratio (MMR) of 420 per 100,000 live births or greater as of 2015 [1, 2]. The overwhelming 80-fold difference in the estimated lifetime risk of maternal mortality in low-income countries, as compared to high-income countries, points to the diligence of insightful inequality that must be addressed [3]. Inequalities in access to care particularly for vulnerable populations; poor quality of available care; grave deficiencies in health system infrastructure and workforce; and the impact of economic, political, socio-demographic and environmental factors all contribute significantly to the risk of poor maternal health outcomes and hinder progress toward reduction in mortality and morbidity [4–9]. In this situation, attaining the sustainable development goal (SDG) will not be possible without reducing the burden of the deprived in all population subgroups.

Nepal has made remarkable progress with respect to improving the situation of maternal health in the last two decades [10, 11]. Percentage of pregnant women with at least four antenatal care (ANC) visits has increased from 14% in 2001 to 69% in 2016. Similarly, more women (58%) were assisted during delivery by a skilled provider in 2016 compared to the situation in 2001 (11%) [12]. Though the government is committed to promoting equity in distribution and utilization of health services through its health policies and sector strategies, the studies have found the unfair distribution of benefits from public investment on health across different population sub-groups [11, 13]. Inequitable access to and utilization of maternal health services due to financial, socio-cultural, and geographical barriers, among others are key challenges [14].

Maternal health has been one of the high priority programs of the Government of Nepal (GoN). The Government aims to reduce the maternal mortality ratio to 70 per 100,000 live births by 2030 as part of the SDGs target from 239 in 2016 [15]. To improve the situation of maternal health, GoN has implemented various demand and supply-side interventions. Regarding demand-side intervention, the maternity incentive scheme was initiated in 2005 that made a provision to pay a fixed amount to mother/family to help with the cost of transportation. In 2009 the benefit under the scheme was broadened and the user fee associated with all types of delivery care was removed (Aama program) [13]. Further, in 2012 incentives for completing four ANC with the recommended schedule followed by institutional delivery (introduced in 2009 as separate demand-side financing) was added to the Aama program [16, 17]. Regarding supply-side interventions, extensive expansion of service delivery sites was made through the establishment of health posts and sub-health posts throughout the country following the endorsement of National Health Policy 1991. The Safe Motherhood Policy and Plan of Action (1994–1997); National Safe Motherhood Policy 1998; National Safe Motherhood and Newborn Health Long Term Plan (2006–2017); and National Policy on Skilled Birth Attendants (2006) introduced key policy interventions regarding the availability of maternal health care in rural and remote areas. The examples of such interventions are: establishment of birthing centers (BCs) in health posts, establishment of emergency obstetric centers (EOC) in primary health care centers and district hospitals, availability of skilled birth attendant training to nursing staffs and doctors of BCs and EOCs in training sites, and the strengthening of the referral services.

The National Health Policy 2019 envisions equitable access to healthcare with respect to all the population groups and emphasizes on providing a high priority to the poor and vulnerable [18]. Similarly, Nepal Health Sector Strategy (NHSS) (2015–2020) has recognized inequity as one of the key challenges of the health sector in Nepal [14], though the government has implemented special incentives such as free health care programme and safe delivery incentive scheme with an objective to reduce inequity in healthcare utilization. Further, equity and access are identified as one of the four strategic pillars by NHSS to move towards universal health coverage, other three being: quality, health sector reform and multi-sectoral collaboration. Nepal Health Sector Plan [19, 20] has planned to strengthen the health system so that the poor and vulnerable communities have the priority for access. National Health Policy 1991 established primary health care in Nepal (sub-health post in every ward) and provided special emphasis to the population of rural areas [21]. SDGs in Nepal, structured on three dimensions: economic, social and environment, are set with a vision of transforming Nepal into a prosperous nation with equity and social justice [15]. With all these efforts and many others, the equity gap in accessing and using maternal health in the general population has decreased over the period of 2001 to 2016 as presented by different papers [11, 13]. In this paper we have analyzed whether the equity gap in utilization of key maternal health services has decreased across seven provinces over this period as the geography of Nepal has already been identified as one of the predictors of availability, access and utilization of maternal health services [13, 14]. The constitution of Nepal promulgated in 2015 has institutionalized Nepal as a Federal Democratic Republic country with seven provinces. Evidence on the status of equity gap and progress over time related to maternal health indicators across the provinces (subnational) will help policymakers and planners at the federal and provincial level equitably allocate scarce resources. Therefore, it is high time we measure whether the distribution of health indicators at the sub-national level is equitable and provide evidence to pursue the agenda of Universal Health Coverage (UHC) at different level.

## Methods

### Data source and sampling design

**Fig. 1:**
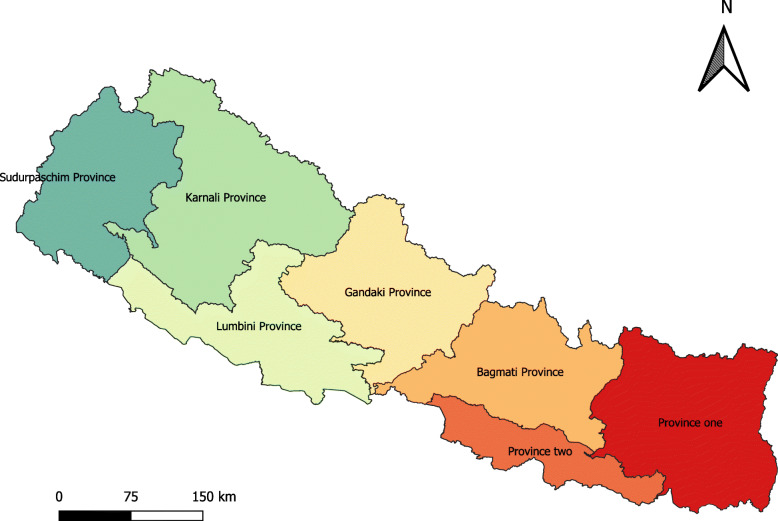
Map of Nepal with provincial boundaries

This study is based on the data from Demographic and Health Surveys (DHS) implemented by DHS program [22] which collects data on fertility, family planning, health and nutrition, health services utilization, health knowledge and behaviors from adult men and women from serial surveys in over 80 Low and Middle Income Countries (LMICs). Trained interviewers collected data in separate standardized questionnaires from eligible adult women (15–49 years) and men (15–49 years) living in the sampled households through face-to-face interviews during in-home surveys. The DHS sample is selected in multiple stages. The first stage involves selecting clusters with probability proportional to size from a national master sample frame. In next stage, a systematic sample of households is drawn from a listing of households in each of the DHS clusters. While the sample of Nepal Demographic and Health Survey (NDHS) 2001, 2006, and 2011 were selected in two stages, the sample of NDHS 2016 was stratified and selected in two stages in rural areas and three stages in urban areas. Details of the DHS design and methodology are described elsewhere [12, 23]. We used one primary stratification variable for assessing inequalities: Province. We showed inequalities by provinces because the new constitution of Nepal endorsed in September 2015, devolved the power into three levels of government: one federal level, seven provinces, and 753 local governments [24]. Recently conducted NDHS 2016 stratified each province into urban and rural areas, yielding 14 sampling strata. For the provinces of the previous surveys, we have verified the comparability of clusters across each of the surveys using Global Positioning System (GPS) code of the clusters [25]. For instance, the GPS of each cluster were used for the identification of the districts hence the districts were used for the identification of provinces in earlier surveys. The map of Nepal with provincial boundaries is shown in the Fig. 1. We did not include the Nepal Family Health Survey of 1996 because we could not locate verified clusters. Therefore, only four rounds of DHS in Nepal were included in the analysis (2001–2016). Due to the small number of cases, only three rounds of DHS (2006–2016) were used in the analysis for Caesarean Section (CS) delivery for Karnali Province.

### Selection of variables

A review of literature disclose that Nepal has largely taken an identical approach to delivering maternal and child health (MCH) services [11]. While MCH services are priority in a health sector in Nepal, attention has been focused on attaining MCH related targets with programs and interventions established in easily accessible areas and without due consideration to the disparities that affect access to services. In other words, efforts to improve national level maternal health indicators until now have often overlooked subnational and inequalities [26]. It is therefore crucial that inequalities of MCH service use, and the amount and nature of inequalities are examined and implicit, so that strategies and programs to address inequities can be developed in subnational level. Hence, our outcome variable is utilization of maternal health services: At least four ANC, Institutional delivery, and CS delivery. CS delivery was also selected for analysis because it is one of the important indicators of utilization of Comprehensive Emergency Obstetric Care (CEOC). Operational definitions of these maternal health service indicators are given below.

#### Four ANC

Percentage of women aged 15–49 years who had a live birth in the five years preceding the survey that received four or more antenatal check-ups.

#### Institutional delivery

Percentage of live births in the five years preceding the survey delivered in a health facility (private or public).

#### CS delivery

Percentage of live births in the five years preceding the survey delivered by caesarian section in a health facility (private or public).

The study calculated summary statistics (percent and CI) of these indicators for each survey round and across the provinces weighted by individual sample weights.

### Inequalities measurements

For the equity analysis, the study used the wealth index scores for each household. The wealth index score was calculated based on a household’s ownership of selected assets, such as televisions and bicycles; materials used for housing construction; and access to water and sanitation facilities and the scores are comparable in different rounds of surveys [27–29]. This paper uses concentration curves and indexes in its relative formulation, with no corrections. The concentration index is defined as twice the area between the concentration curve and the line of equality (the 45-degree line) and was calculated adopting the procedure described by O’donnell, Owen, et al. [30]. Concentration curves are used to identify socioeconomic inequality in maternity services and whether it is more pronounced at one point in time than is the other or in one province than another. The concentration index depends only on the relationship between the health variable and the rank of the living standards variable and not on the variation in the living standards variable itself. A change in the degree of income inequality does not affect the concentration index measure of income-related health inequality. Therefore, it can be used to compare the change in inequality over the time period. It can be computed from microdata by using the “convenient covariance” eq. 1 as shown below.

1 $$ C=\frac{2}{\mu}\mathit{\operatorname{cov}}\left(h,r\right) $$

Where *h* is the health sector variable, *μ* is its mean, and *r* = *i*/*N* is the fractional rank of individual *i* in the living standards distribution, with *i* = 1 for the poorest and *i* = *N* for the richest.

The survey is not self-weighted, so the weights were applied in computation of the covariance, the mean of the health variable, and the fractional rank. Given the relationship between covariance and fractional rank using ordinary least squares (OLS) regression, an equivalent estimate of the concentration index is obtained from a “convenient regression” of a transformation of the health variable of interest on the fractional rank in the living standards distribution [31], as shown in eq. 2.

2 $$ 2{\sigma}^2\left(\frac{h_i}{\mu}\right)=\alpha +\upbeta {r}_i+{\varepsilon}_i $$

where *σ*^2^ is the variance of the fractional rank. The OLS estimate of *β* is an estimate of the concentration index equivalent to that obtained from eq. 1. This method gives rise to an alternative interpretation of the concentration index as the slope of a line passing through the heads of a parade of people, ranked by their living standards, with each individual’s height proportional to the value of his or her health variable, expressed as a fraction of the mean. Test of significance of changes in concentration index was performed by calculating pooled standard errors of change in concentration index [32].

The estimation and analysis were performed in R software for statistical computing [33]. The strata for the survey design were different for the 2016 survey as compared to the previous surveys; and our objective was to estimate the inequality at the provincial level, strata were not used in the survey design during the estimation process. In order to ensure the comparability of sampling design in analysis, the estimation process did not consider the strata in the survey design. The survey [34] package in R was used to take into account the complex survey design. The concentration curves were prepared using the *ggplot2* package in R [35].

### Ethical consideration

The ethical clearance for these surveys was obtained by institutional review board of ICF/DHS program and Nepal Health Research Council (NHRC). Informed consent was sought from each survey participant as per the international guidelines. Since this analysis was based on publicly available data, no further ethical permission was necessary. Administrative permissions for data were required and obtained from the DHS program (https://dhsprogram.com/Data/terms-of-use.cfm).

## Results

**Fig. 2:**
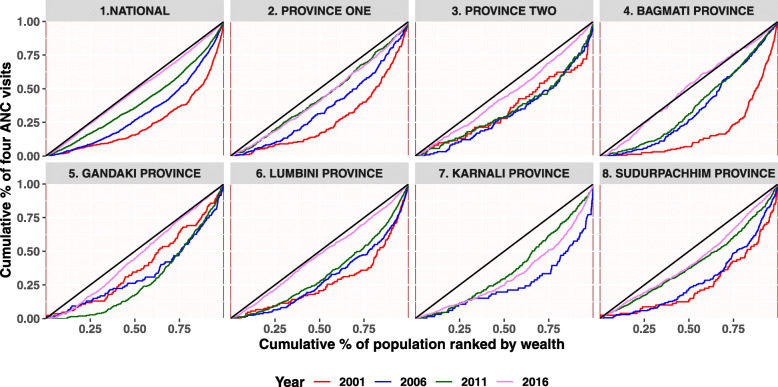
Concentration Curve for at least four ANC visits

**Fig. 3:**
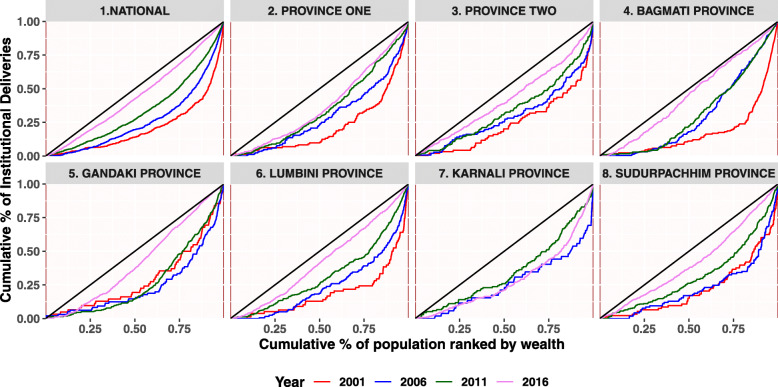
Concentration Curve for Institutional Delivery

**Fig. 4:**
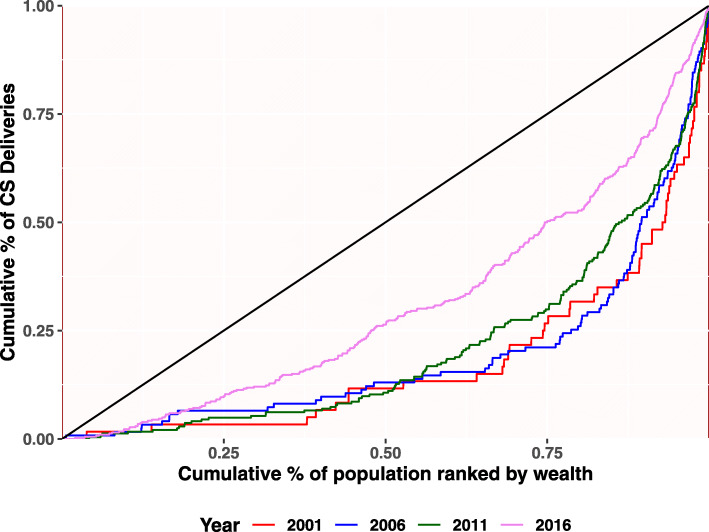
Concentration Curve for CS delivery

This section begins with a descriptive analysis of the variables used in the analysis. Table 1 provides the national and provincial level statistics for the three indicators. Figures 2, 3 and 4 and Table 2 provides detailed findings on concentration curves and indexes disaggregated by the provinces. Finally, Table 3 ends with an explanation of temporal changes in concentration index at the national and provincial level.

**Table 1:** Distribution of at least four ANC, institutional delivery and CS delivery by province

	Survey year							
	2001 n(%)	95%CI	2006 n(%)	95%CI	2011 n(%)	95%CI	2016 n(%)	95%CI
**At least four ANC**								
**National**	**4735 (14.3)**	**12.1–16.6**	**4065 (29.5)**	**25.8–33.1**	**4148 (50.1)**	**46.0–54.2**	**3998 (69.4)**	**66.4–72.3**
Province one	855 (22.3)	16.7–28.0	687 (32.3)	25.9–38.7	910 (52.7)	44.8–60.6	686 (76.9)	71.8–82.0
Province two	1067 (8.2)	5.2–11.2	818 (18.1)	13.2–23.0	810 (33.5)	25.0–42.0	963 (53.4)	47.2–59.6
Bagmati Province	707 (20.6)	12.6–28.6	708 (43.9)	33.6–54.1	572 (60.7)	50.7–70.7	691 (78.4)	71.7–85.1
Gandaki Province	450 (22.3)	13.7–31.0	424 (26.8)	17.0–36.6	479 (53.0)	40.0–66.1	337 (76.7)	69.6–83.7
Lumbini Province	764 (13.4)	8.8–18.1	605 (29.9)	22.3–37.4	654 (53.2)	44.2–62.2	720 (73.7)	67.1–80.3
Karnali Province	392 (3.1)	−0.6-6.8	240 (18.3)	9.1–27.5	283 (39.9)	28.4–51.4	255 (52.2)	44.4–60.1
Sudurpaschim Province	501 (7.9)	4.2–11.6	583 (30.6)	17.4–43.8	440 (60.2)	53.0–67.5	346 (77.3)	72.7–81.9
**Institutional delivery**								
**National**	**6972 (9.1)**	**7.4–10.7**	**5545 (17.7)**	**14.8–20.5**	**5391 (35.3)**	**31.8–38.9**	**5060 (57.4)**	**53.8–60.9**
Province one	1243 (11.4)	7.6–15.3	920 (17.4)	12.3–22.6	1135 (41.4)	33.7–49.1	819 (62.2)	55.2–69.3
Province two	1638 (7.7)	4.6–10.8	1171 (13.1)	8.6–17.6	1146 (28.6)	22.0–35.2	1367 (44.6)	37.9–51.3
Bagmati Province	1036 (15.3)	8.6–22.0	920 (35.1)	25.0–45.1	705 (45.1)	32.5–57.6	813 (70.7)	61.3–80.1
Gandaki Province	602 (12.3)	6.8–17.9	571 (17.6)	8.9–26.3	589 (42.6)	30.2–55.0	388 (68.3)	57.9–78.7
Lumbini Province	1090 (6.6)	3.9–9.3	822 (16.2)	11.3–21.2	810 (34.6)	26.5–42.6	899 (59.4)	52.0–66.7
Karnali Province	617 (2.1)	1.1–3.1	341 (12.1)	1.0–23.1	401 (20.7)	13.2–28.2	338 (35.6)	26.1–45.2
Sudurpaschim Province	747 (6.2)	3.4–8.9	800 (8.5)	5.1–12.0	605 (29.0)	22.0–35.9	437 (66.4)	58.3–74.5
**CS delivery**								
**National**	**6972 (0.8)**	**0.5–1.1**	**5545 (2.7)**	**1.9–3.5**	**5391 (4.6)**	**3.6–5.6**	**5060 (9.0)**	**7.5–10.5**
Province one	1243 (0.8)	0.1–1.4	920 (2.1)	0.8–3.5	1135 (6.5)	3.5–9.5	819 (12.7)	9.2–16.2
Province two	1638 (0.8)	0.3–1.4	1171 (2.1)	0.7–3.5	1146 (4.1)	2.9–5.3	1367 (5.0)	2.8–7.3
Bagmati Province	1036 (2.1)	0.8–3.3	920 (6.0)	2.7–9.3	705 (8.3)	4.3–12.4	813 (17.4)	11.9–22.8
Gandaki Province	602 (1.4)	−0.3-3.2	571 (1.7)	− 0.1-3.5	589 (4.0)	1.7–6.4	388 (16.7)	12.3–21.0
Lumbini Province	1090 (0.2)	0.0–0.5	822 (3.3)	1.5–5.1	810 (3.7)	1.8–5.6	899 (6.4)	4.3–8.4
Karnali Province	617 (0.0)	0.0–0.0	341 (1.4)	0.0–2.9	401 (1.0)	0.0–2.0	338 (2.2)	0.9–3.4
Sudurpaschim Province	747 (0.3)	0.1–0.6	800 (0.8)	0.1–1.5	605 (1.8)	0.8–2.9	437 (3.1)	1.6–4.5

Note: *n* indicates the total number of respondents in each province and figure in brackets indicate percent

**Table 2:** Relative concentration index of at least four ANC, institutional delivery and CS delivery by province

	2001	95%CI	2006	95%CI	2011	95%CI	2016	95%CI
**At least four ANC**								
**National**	**0.46**	**0.39–0.52**	**0.32**	**0.27–0.36**	**0.22**	**0.19–0.24**	**0.08**	**0.07–0.10**
Province one	0.40	0.33–0.46	0.31	0.24–0.39	0.20	0.15–0.24	0.06	0.03–0.09
Province two	0.28	0.09–0.47	0.29	0.15–0.43	0.22	0.12–0.32	0.09	0.04–0.13
Bagmati Province	0.58	0.45–0.71	0.33	0.26–0.39	0.26	0.21–0.32	0.10	0.06–0.13
Gandaki Province	0.20	0.06–0.35	0.26	0.16–0.36	0.30	0.24–0.35	0.13	0.09–0.17
Lumbini Province	0.43	0.30–0.57	0.33	0.25–0.41	0.22	0.18–0.27	0.05	0.03–0.07
Karnali Province	0.13	−0.07-0.34	0.26	0.13–0.38	0.22	0.10–0.33	0.20	0.14–0.26
Sudurpaschim Province	0.45	0.26–0.64	0.33	0.26–0.40	0.14	0.10–0.18	0.05	0.01–0.08
**Institutional delivery**								
**National**	**0.56**	**0.46–0.66**	**0.48**	**0.42–0.55**	**0.35**	**0.32–0.38**	**0.19**	**0.16–0.21**
Province one	0.53	0.40–0.65	0.48	0.36–0.59	0.32	0.28–0.37	0.21	0.17–0.26
Province two	0.49	0.25–0.73	0.36	0.18–0.54	0.21	0.12–0.30	0.13	0.08–0.19
Bagmati Province	0.63	0.43–0.83	0.45	0.39–0.52	0.43	0.36–0.50	0.21	0.17–0.25
Gandaki Province	0.40	0.21–0.58	0.48	0.33–0.62	0.34	0.29–0.39	0.21	0.17–0.26
Lumbini Province	0.56	0.31–0.81	0.46	0.33–0.58	0.33	0.26–0.39	0.15	0.10–0.20
Karnali Province	0.37	−0.11-0.85	0.23	0.06–0.41	0.30	0.15–0.44	0.32	0.24–0.39
Sudurpaschim Province	0.52	0.30–0.73	0.38	0.16–0.60	0.34	0.27–0.42	0.12	0.06–0.17
**CS delivery**								
**National**	**0.62**	**0.36–0.88**	**0.62**	**0.41–0.83**	**0.54**	**0.42–0.67**	**0.44**	**0.35–0.53**
Province one	0.63	0.08–1.19	0.63	0.14–1.12	0.41	0.20–0.62	0.39	0.25–0.53
Province two	0.40	−0.07-0.86	0.61	0.15–1.08	0.15	−0.11-0.42	0.07	−0.10-0.23
Bagmati Province	0.64	0.24–1.04	0.66	0.44–0.89	0.71	0.41–1.00	0.52	0.38–0.66
Gandaki Province	0.48	−0.15-1.10	−0.15	− 0.97-0.67	0.61	0.33–0.89	0.32	0.22–0.42
Lumbini Province	0.36	−0.31-1.04	0.69	0.32–1.05	0.59	0.31–0.86	0.49	0.28–0.71
Karnali Province	–	–	0.43	−0.48-1.34	0.62	−0.16-1.40	0.52	0.18–0.86
Sudurpaschim Province	0.83	0.24–1.43	0.39	−0.13-0.90	0.55	0.27–0.83	0.42	0.17–0.67

**Table 3:** Time wise differences of relative concentration index of at least four ANC, institutional delivery and CS delivery by province

	Relative concentration index in 2001	Difference between 2001 and 2006	Difference between 2006 and 2011	Difference between 2011 and 2016
**At least four ANC**				
**National**	**0.46**	−0.136**	− 0.103***	−0.135***
Province one	0.40	−0.087	−0.117**	− 0.136***
Province two	0.28	0.008	−0.067	−0.134*
Bagmati Province	0.58	−0.255**	−0.061	− 0.166***
Gandaki Province	0.20	0.058	0.036	−0.169***
Lumbini Province	0.43	−0.106	−0.105*	− 0.171***
Karnali Province	0.13	0.121	−0.038	−0.017
Sudurpaschim Province	0.45	−0.122	−0.186***	− 0.094**
**Institutional delivery**				
**National**	**0.56**	**−0.077**	−0.132***	− 0.163***
Province one	0.53	−0.049	−0.151*	− 0.112**
Province two	0.49	−0.130	−0.150	− 0.076
Bagmati Province	0.63	−0.177	−0.022	− 0.220**
Gandaki Province	0.40	0.081	−0.138	−0.127***
Lumbini Province	0.56	−0.102	−0.130	− 0.177***
Karnali Province	0.37	−0.138	0.065	0.018
Sudurpaschim Province	0.52	−0.138	−0.035	− 0.226***
**CS delivery**				
**National**	**0.62**	**0.001**	**−0.079**	**−0.099**
Province one	0.63	−0.003	−0.217	− 0.024
Province two	0.40	0.217	−0.459	−0.085
Bagmati Province	0.64	0.023	0.041	−0.187
Gandaki Province	0.48	−0.627	0.757	−0.289
Lumbini Province	0.36	0.323	−0.097	−0.095
Karnali Province	–	–	0.193	−0.101
Sudurpaschim Province	0.83	−0.449	0.165	−0.131

***p < 0.001, **p < 0.01,*p < 0.05

Note: The * are based on the *p* values for the test of significance of difference between the concentration index between two consecutive years

### Descriptive analysis

The proportion of women with at least four ANC visits for most recent birth in the five years before the survey increased by 55-percentage-point from 2001 NDHS to 2016 NDHS. In 2001–2016, every province has a large and steady increase in the proportion of women with at least four ANC visits. The increasing trend is found to be higher in Sudurpaschim Province (69-percentage-point) followed by Lumbini Province (60-percentage-point) and Bagmati Province (58-percentage-point). Similarly, the percent of births in the five years preceding the survey delivered in health facilities increased by two-fold in 2006 compared to 2001 and doubled again to 35 percentages in 2011. Similarly, between 2011 and 2016, there was a noteworthy 22- percentage-point increase in the proportion of institutional deliveries. Similar to four or more ANC visits, all provinces have huge and stable increases in the proportion of institutional delivery. Further, the proportion of births delivered by CS has increased from one percentage in 2001 to nine percentage in 2016. Notably, Province one (from 0.8 percentage to 12.7 percentage), Bagmati Province (from 2.1 percentage to 17.4 percentage) and Lumbini Province (from 1.4 percentage to 16.7 percentage) have largely increased the proportion of CS delivery in the years between 2001 and 2016. The Karnali and Sudurpaschim Provinces have non-existent and very low coverage of CS delivery respectively at the beginning of the survey. In subsequent years the coverage has increased gradually, still remarkably lower than the situation in other provinces (Table 1).

### Measures of inequality: concentration curve and index

This section covers measures of inequality in terms of concentration curves and indexes.Inequality is explained in terms of a set of concentration curves for each survey year and across each province. The 45-degree line is the egalitarian line also called the line of equality. The curves are interpreted with reference to the 45-degree. The figure includes eight parts-one for each province starting with the national aggregate. Similarly, four rounds of survey findings are also covered in the diagram.

Figure 2 shows how the situation of inequality for at least four ANC visits has changed over different survey periods at national and subnational level. At national level, the curves are below the line of equality indicating that the coverage is disproportionately higher among the rich women, however, it is shifting towards the 45-degree line over the period of fifteen years. The shift in curve is more pronounced while moving from 2011 to 2016. In Province one, the curves for different years are gradually moving towards the line of equality from 2006 to 2011, however subsequent movement cannot be observed. Similarly, in Province two, the situation of inequality has worsened from 2001 to 2006 and remained almost the same until 2011, with only improvement observed while moving from 2011 to 2016. Likewise, in Bagmati Province there is a remarkable reduction in inequality between 2001 and 2006, however no marked improvement between 2006 and 2001. However, the situation has profoundly improved in 2016, the curve almost overlapping with the line of equality. In Gandaki Province the situation of inequality is deteriorating from 2001 to 2011, however the status of equality has remarkably improved during the time period of 2011 and 2016. In Lumbini Province, the inequality has gradually decreased over the different survey period, a marked decrease observed between 2011 and 2016. In Karnali Province, the situation of inequality has markedly decreased between 2006 and 2011, however, further worsened in 2016. In Sudurpaschim Province the inequality has gradually decreased over different survey periods, remarkable change can be observed between 2006 and 2011.

Figure 3 shows how the situation of inequality for institutional delivery changed over different survey periods at national and subnational level. At national level, the curves are below the line of equality indicating that the coverage is disproportionately higher among the rich women, however, it is shifting towards the 45-degree line over the period of fifteen years. The shift in curve is more pronounced while moving from 2011 to 2016. In Gandaki Province the interlocked curves for survey years 2001 to 2011 signifies no major changes in the status of inequity over these years, however, a slight movement of curves towards the line of equality is observed in 2016. Consistent movement of curves towards the line of equality is observed in Province one, Province two, Bagmati Province, Lumbini Province and Sudurpaschim Province (only for 2011 and 2016). In Karnali, all the curves are far from the line of equality, indicating meagre progress in addressing inequality.

Figure 4 shows how the situation of the concentration covers for CS delivery changed over different survey periods at national level. The inequality is very high in the beginning. The interlocked curves of survey years 2001 to 2011 indicates lack of improvement in the status of inequality over this period, signifying wealthy households disproportionately benefited from CS delivery. However, a pronounced shift in the curve was observed from 2011 to 2016.

The concentration index provides a summary measure of the magnitude of socioeconomic-related inequality. Along with concentration indexes, we have also presented the concentration curves to assess the disparities and their changes among the women. Table 2 shows that concentration index has positive values which implies that the benefits were more concentrated in the higher wealth quintiles than the lower. Table 2 shows concentration index by national and subnational , over the period of time for at least four ANC, institutional delivery and CS delivery. Overall, at national level, relative inequalities of at least four ANC progressively decreased from 2001 (RCI = 0.46; 95% CI = 0.39–0.52) to 2016 (RCI = 0.08; 95% CI = 0.07–0.10). Similarly, the relative concentration index of institutional delivery also gradually decreased from 0.56 (95% CI: 0.46–0.66) to 0.19 (95% CI: 0.16–0.21) in four rounds of the survey. The relative inequalities of CS delivery, however, did not decrease as steadily as compared to that of at least four ANC and institutional delivery. Further, all the provinces have decreased the relative concentration index for four or more ANC overtime except Karnali Province. Province two progresses fairly well but only between 2011 and 2016. Similarly, institutional delivery has decreased the relative concentration index overtime with a lower rate of decrement in Karnali Province. Except Lumbini and Karnali Province, CS delivery has also decreased relative concentration index overtime. The concentration curve (Fig. 2 and Fig. 3) ratifies these findings, as the curve has shifted distally to the line of equality for maternal health services except in Karnali Province. Unlike other years, Karnali Province still has higher inequality than other provinces (concentration index of at least four ANC = 0.20, concentration index of institutional delivery = 0.32 and RCI of Cs delivery = 0.52).

### Inequality over the time: concentration index

Table 3 shows changes in concentration index between 2001 and 2016 for the three indicators. A progressive and statistically significant decrease in concentration index was observed for at least four ANC visits at national level. The change between 2001 and 2016 was also disaggregated across the provinces. Only Bagmati Province witnessed a huge and significant decrease in inequality. For the rest of the provinces, the changes are modest and are not statistically significant. Between 2006 and 2011, Province one, Lumbini and Sudurpaschim Province witnessed a statistically significant decrease in concentration index and among them, the greatest decrease in inequality was achieved by Sudurpaschim Province. Similarly, the changes between 2011 and 2016 are statistically significant and progressive at aggregate and provincial level. Bagmati Province, Gandaki Province and Lumbini Province were found to progress mostly while the Karnali Province was still lagging behind in decreasing the inequality.

At the aggregate level, inequality in institutional delivery was observed to be decreasing appreciably between 2001 and 2016 and the greatest decrease was observed between 2011 and 2016. In the early years, however, the decrease in inequality at national level was not observed to be statistically significant. Similarly, none of the provinces exhibited a significant decrease in concentration index either. The changes in the index between 2006 and 2011 were showing a statistically significant decreasing trend. At the subnational level during this period, only province one showed a significant decrease while in the rest of the provinces, the decrease was not statistically significant. The provinces, however showed a statistically significant decrease in concentration index between 2011 and 2016 except for the Karnali Province. During the same period, Bagmati Province, Lumbini Province and Sudurpaschim Province made the highest progress in decreasing inequality in institutional delivery.

For CS delivery, the concentration index decreased over the period of 2001 and 2016. However, the changes were not statistically significant. Similarly, none of the changes, even at the provincial level, between the period of 2001 and 2016 were found to be statistically significant though the curve is shifting towards the line of equality over the period from 2001 to 2016(Fig. 4) and the greatest shift in the curve was observed in the year of 2016.

## Discussion

The main aim of this study was to find the trends and inequalities at national and subnationall level with respect to the use of maternal health services in Nepal using the nationally representative cross-sectional study (Demographic and Health Survey) over the period of 2001 to 2016. In general, the study found that the status of inequality in utilization of maternal health services in Nepal depicted by three indicators (at least four ANC visits, Institutional delivery and CS delivery) has improved remarkably over the survey period. However, the progress is not proportional across seven provinces. Similar findings were obtained in other studies based on NDHS data [11, 13].

The increase in the uptake of maternal health services during this period, such as institutional delivery, has been reported to be associated with the mix of supply and demand side investments made by the GoN [17]. Strengthening of the healthcare delivery system (supply side) specifically since the late 1990s (Safe motherhood long term plan) has made maternal health more accessible to the poor and women from hard to reach geography [36]. The example includes the construction and expansion of birthing centers (BCs) in most disadvantaged areas, strengthening of BEOC/CEOC, production of auxiliary nurse midwives (ANMs) and training of skilled birth attendant (SBA to nursing staffs and advanced SBA to medical officers), training of nurse-midwives as anesthetic assistants and strengthening of blood banking [17, 36, 37]. Similarly, Demand Side Financing (DSF) policy in maternal health, introduced first in 2005 and revised in subsequent years, is also reported to be associated with the increase in access and utilization of maternal health care in Nepal [17]. Studies conducted in other low and middle-income countries (India, Bangladesh) to understand the effect of demand side financing on maternal health outcomes has shown that cash incentives can contribute in increasing the utilization of ANC and institutional delivery services [38, 39]. The targeted voucher programs implemented in Cambodia and Pakistan showed improved access to institutional deliveries by poor women and thus reducing inequalities in utilization [40, 41]. An assessment conducted by the Ministry of Health and Population has shown that demand side financing in Nepal was associated with an increase in uptake of maternal health services such as institutional delivery [17, 42]. Similarly, another analysis has shown that the demand side financing in maternal health had a major effect on utilization of at least four ANC and institutional delivery [16].

With respect to the use of at least four ANC visits, we observed that the concentration curves are gradually moving towards the line of equality with each subsequent survey period (2001, 2006, 2001, 2016). The shift towards the line of equality is more prominent between the survey period of 2011 and 2016. The movement towards equality is progressive and statistically significant. The combined effect of both supply and demand side interventions in maternal health can be attributed to such a change. Talking about the Provinces, somehow consistent improvement in the status of inequality with respect to the use of at least four ANC services was observed in Province one, Bagmati Province, Lumbini Province and Sudhurpaschim Province. Bagmati Province has witnessed a huge and significant decrease in inequality, particularly during the survey period 2001–2006 and 2011–2016. Similarly, Province one, Lumbini and Sudurpaschim have witnessed a statistically significant decrease in concentration index between 2006 and 2011. Particularly, statistically significant and progressive change is observed at both national and provincial level during the survey period of 2011–2016, however, Karnali province and Province two are comparatively lagging behind. The studies have also shown that the benefits of DSF policies are skewed towards areas that are comparatively better off in terms of wealth and geography [17], such as Province one, Bagmati Province and Lumbini Province that are more accessible and wealthy. In contrast, the women from mountain areas (Karnali Province) have found to be comparatively least benefited from the DSF interventions [43]. So the finding in our study can be linked with earlier studies that indicated limitation of DSF to sufficiently address geographical remoteness and poor transport links [41, 44–54].

With respect to the institutional delivery, the greatest decrease in inequality was observed between 2011 and 2016 at the national and subnational level, except for Province two and Karnali. It has been shown that due to lack of various supply side factors such as birthing center services, trained health workers and lifesaving surgical provision in hard-to-reach geography (high hill and mountain area of Nepal) the progress towards the utilization of maternal health services is relatively poorer compared to more accessible areas [17]. It is also argued that since the health facilities in these Provinces have to be accessed only with hours (in some cases days) of walking on foot due to lack of transport facilities, the opportunity cost associated with this plays a crucial part in reducing the demand [55]. Though Province two is plain, this province is one of the poorest with respect to the overall socio-economic status [56, 57]. So, available evidence supports our finding that the progress in addressing the level of inequality is sluggish in Karnali Province and Province two. Bagmati Province, Lumbini Province and Sudurpaschim Province have made comparatively better progress towards reducing inequality, which is as expected because these are comparatively better off regions in terms of socio-economic development and geographical positioning [56, 57]. There was no significant improvement in the level of inequality between 2001 and 2006, even at the national level. Possible reasons could be that the DSF in maternal health was started in 2005 and rapid maternal health service expansion through BCs/EOCs accompanied with SBA training was started after 2006. Similarly, Safe Motherhood and Neonatal Health Long Term Plan (2006–2017) initiated after 2006 and associate interventions at community level that focused on birth preparedness and institutional delivery could also explain this finding. However, some improvement was observed during 2006–2011 at national and provincial level (Province one only).

With respect to CS delivery, the concentration index is observed to be decreased over the period of 2001–2016 at national level, however, the changes are not statistically significant. Similarly, the curves are shifting towards the line of equality over the period between 2001 and 2016, a greater shift observed while moving from 2011 to 2016. Similar study using DHS data found an increase in concentration index over this period of survey years [11]. A study conducted in 2016 in western Nepal shows that odds of going through CS delivery was four times more in women from urban areas compared to that of rural areas [58]. Similarly, the likelihood of using CS delivery was found 10 times more in women from the richest quintile compared to poorest [11]. A study from a tertiary hospital in Kathmandu reports a dramatic rise in CS rates, from around 21% in 2004 to 39% in 2014 [59].

Disproportionate in status of utilization of maternal health services and unequal progress towards equality could be linked to various factors. One such factor, as pointed out in other papers as well, is that the Government’s blanket approach in delivering maternal health services across the country [60], with greater emphasis on achieving national targets and less attention to monitor subnational targets and equality [26]. An example is the universal nature of demand side financing in maternal health in Nepal that aims to cater the maternal health care needs of all without specific focus on the women that have barriers due to geographic and socio-economic factors, such as women form Province two and Karnali Province. This also shows that the investment of government in maternal health including the DSF initiatives of GoN could not address the geographical, financial and social barriers present differently across seven provinces [38]. Second is linked with the difference in level of availability and accessibility of health facilities in different provinces (supply side investment). A national level survey on availability and readiness of health services shows that the percentage of facilities providing normal vaginal delivery is lowest in Province two, whereas one of the lowest percentages of health professionals are trained for normal delivery in Karnali Province. Province two has the lowest percentage of health professionals trained for ANC [61]. So the gaps in the situation of supply side investment in different provinces witnessed in terms of availability of services, essential medicines and readiness (in terms of skill, equipment, medicine) may explain the inequality in service utilization [61]. Another study using the nationally representative health facility survey showed that the mean general health service readiness score was lowest in Province two [62]. Third is the underlying socio-economic and geographical position of the provinces. The Human Development Index in 2011 was lowest of Karnali Province (0.475), followed by Suderpaschim Province (0.478) and Province two (0.485), and the lowest annual change was observed in Province two since 1996 [56]. Fourth is lack of special focus of government towards provinces that are geographically challenged and socio-economically lagging behind (already discussed above). So, these evidence points out towards the need of special strategies from the government side tailored across the provinces.

The study has a few limitations that readers should be aware of while interpreting findings. First, this study describes the maternal health services inequalities at national and provincial level and does not take any predictors of inequalities in maternal health services into account. However, the study discussed the observed changes in inequalities in light of government policies during that period. Second, the analysis covers multiple rounds of cross-sectional surveys to analyze the trend in inequality. However, in the survey round of 2016, the strata were different from that of previous surveys. For the comparison purpose, we did not take into account the strata in survey design analysis. This generally affects the standard error of the revised estimates but the approach ensured comparability of methods employed across the survey rounds. Third, the frequency distribution of some of the indicators across provinces is very low in the year of 2001 and 2006. This might be the reason for statistically non-significant results despite a noticeable shift in concentration curve. These limitations, however, do not dilute the relevance of the findings for the policy makers. Fourth, the frequency distribution of CS delivery is very low when stratified across the provinces. Therefore, study reported the concentration curves at national level only. Fifth, the frequency distribution of indicators are very low for Karnali Province in 2001. We therefore could not produce a meaningful concentration curve for Karnali Province for 2001. Despite these limitations, the study reports observed trends in inequalities at national and subnational level, which can be useful for policy makers at both levels. The study has observed a narrowing of inequality in use of CS services. However, the complete analysis also requires the systematic analysis of the fact that CS are effective in saving maternal and infant lives only when they are required for medically indicated reasons. Such analysis requires the Robson classification at admission [63] for the pregnancy cases which is not available in the current dataset. However, inequality in utilization of CS services itself reflects useful information to the policy makers for taking pro-poor actions to bridge the gap.

## Conclusion

The study has found a remarkable improvement in the distribution of maternal health indicators across wealth over four consecutive DHS surveys in Nepal. The inequality in utilization of maternal health services among different wealth quintiles is observed to be decreasing with every subsequent survey at national level. However, if the statistics are disintegrated at the subnational level, the progress towards decreasing inequality in utilization of maternal health services among different wealth quintiles is not the same across different provinces; the progress is also not uniform across different survey years. Provinces which are comparatively disadvantaged in terms of underlying factors such as geography and socio-economic status (Province two, Karnali Province) have made minimal to zero progress towards reducing inequality in comparison to other provinces, particularly Province one, Lumbini Province and Bagmati Province. Among the maternal health indicators studied here, CS delivery seems to have made less improvement compared to ANC and institutional delivery. Additional research may be needed to explore the reasons and their implication. Special investment to address barriers to access and utilization in provinces that are lacking to make progress in reducing inequality is urgent. Further studies are needed to understand the strategies required to address the gaps in these provinces and bring about fair improvement.

## Data Availability

We used publicly available data and it is accessible from the DHS program website (https://dhsprogram.com/Data/terms-of-use.cfm) upon the request.

## Abbreviations

ANC: Antenatal care
β: Beta
BEOC: Basic emergency obstetric care
CEOC: Comprehensive emergency obstetric care
CI: Confidence interval
CS: Caesarean section
DHS: Demographic and health survey
DSF: Demand side financing
GoN: Government of Nepal
GPS: Global positioning system
LMICs: Low and middle income countries
MMR: Maternal mortality ratio
NDHS: Nepal demography and health survey
NGO: Non-governmental organization
NHSS: Nepal health sector strategy
NHRC: Nepal health research council
OLS: Ordinary least squares
RCI: Relative concentration index
SBA: Skilled birth attendant
SDG: Sustainable development goal
